# Application of XGBoost Model Optimized by Multi-Algorithm Ensemble in Predicting FRP-Concrete Interfacial Bond Strength

**DOI:** 10.3390/ma18122868

**Published:** 2025-06-17

**Authors:** Yuxin Chen, Yulin Zhang, Chuanqi Li, Jian Zhou

**Affiliations:** School of Resources and Safety Engineering, Central South University, Changsha 410083, China; 245501013@csu.edu.cn (Y.C.); yl_zhang@csu.edu.cn (Y.Z.); chuanqi.li@csu.edu.cn (C.L.)

**Keywords:** FRP-concrete interface, bond strength prediction, Nevergrad, XGBoost model, explainable machine learning

## Abstract

Accurate prediction of fiber-reinforced polymer (FRP)-concrete interfacial bond strength is critical for ensuring the safety of FRP-strengthened structures. This study proposes a predictive model based on extreme gradient boosting (XGBoost), which is enhanced via the Nevergrad optimization framework, to address the limited accuracy of traditional empirical approaches. By integrating seven optimizers from the Nevergrad platform, the model achieves global hyperparameter optimization, and a five-fold cross-validation strategy is employed to improve generalization. The prediction results based on 855 sets of single-lap shear test data demonstrate that the optimized model exhibits significantly superior performance on the test set (R^2^ = 0.9726, RMSE = 1.8745, MAE = 1.3857). Compared to the existing best-performing empirical model, the R^2^ is improved by 22.3%, while the RMSE and MAE are reduced by 63.4% and 61.8%, respectively. SHAP interpretability analysis indicates that the width, thickness, elastic modulus, and bond length of the FRP sheets are the main factors influencing the bond strength prediction. The predictive model developed in this study combines high accuracy with strong interpretability, providing a reliable, intelligent tool for designing FRP-strengthened structures.

## 1. Introduction

Fiber-reinforced polymers (FRP), which are composed of high-strength fibers and a polymer matrix, possess characteristics such as high strength, low weight, and excellent corrosion resistance [1]. As an advanced high-performance composite material, FRP has been widely adopted in civil engineering, particularly for strengthening and repairing reinforced-concrete structures [2,3]. In engineering practice, FRP is typically externally bonded to the surface of concrete, forming an FRP-concrete interface that serves as a critical zone for load transfer. The bond strength at this interface directly affects the composite action of the FRP strengthening system and its overall structural reliability. Therefore, accurately predicting the bond strength at the FRP-concrete interface is essential for engineering design and safety evaluation.

The academic community has systematically investigated the bonding mechanism of the FRP-concrete interface and proposed various bond strength prediction models (Table 1) [3,4,5,6,7,8,9,10,11,12,13,14,15,16,17,18,19,20,21]. Specifically, the proposed approaches fall into three categories: empirical models derived from experimental data, semi-empirical models incorporating fracture mechanics theory, and models based on interfacial bond slip relationships. Early studies relied on single lap shear test data to derive empirical formulations [4,6,8,10]. These formulations typically use average bond shear stress methods to estimate interfacial performance. However, these models often fail to adequately account for critical parameters, such as the effective FRP bond length, material heterogeneity, and localized interfacial effects, resulting in significant deviations when applied to real-world engineering predictions. Subsequent advances in fracture mechanics have enabled the development of semi-empirical bond-strength models [3,5,11,17,18,19], in which parameters such as the effective bond length and stress-field distribution functions have been systematically incorporated. These models can capture the nonlinear bond behavior under load more accurately, thereby providing a more robust theoretical basis for the structural strengthening design. Although these models yield reasonable predictions under certain conditions, their development based on specific experimental datasets limits their applicability and fails to fully account for the complex nonlinear mechanical characteristics of the FRP-concrete interface [22,23,24,25].

In recent years, fueled by the growth of big data and advances in machine learning, new approaches have been developed to address complex nonlinear problems. Recent studies have established diverse predictive models for the FRP–concrete interfacial bond strength using extensive experimental datasets [23,24,25,26,27,28,29,30]. For instance, Zhou et al. [23] employed a backpropagation neural network (BPNN) to develop an explicit prediction model that outperforms most traditional models in terms of accuracy. Su et al. [26] compared three machine learning methods and found that the support vector machine (SVM) exhibited the best performance, with prediction accuracy further enhanced by a stacking ensemble. Zhang et al. [25] systematically evaluated six machine learning models based on a single-lap shear test database, and the results showed that the extreme gradient boosting (XGBoost) achieved a 54% reduction in coefficient of variation compared to optimal traditional models. These studies demonstrate that machine learning methods can substantially improve the accuracy of FRP-concrete interfacial bond strength predictions. However, the generalization ability and stability of these models still require improvement, and the selection of model hyperparameters significantly impacts the final prediction accuracy. Consequently, developing effective hyperparameter optimization frameworks to simultaneously improve prediction precision and enhance model interpretability remains a key research challenge.

This study proposes a Nevergrad-optimized XGBoost framework (Nevergrad-XGBoost) to improve the prediction accuracy and robustness of the FRP-concrete interfacial bond strength. By integrating seven built-in optimizers from Nevergrad for hyperparameter tuning of the XGBoost model, in conjunction with a 5-fold cross-validation mechanism, intelligent optimization of the model’s hyperparameters is achieved to construct the optimal predictive model. Furthermore, to improve model interpretability, the Shapley Additive exPlanations (SHAP) method was employed to quantitatively assess the contributions of different input features, thereby revealing the mechanisms influencing the bond strength at the FRP–concrete interface.

## 2. Database

### 2.1. Data Sources

The data used in this study are derived from a large-scale database established by Zhou et al. [23], which includes 969 sets of FRP–concrete interface single-lap shear test results collected from 34 existing studies. The single-lap shear test is regarded as one of the most effective methods for investigating the bond performance between FRP and concrete interfaces [31]. These tests primarily focus on measuring the ultimate bond strength and encompass the key material properties and geometric parameters. To eliminate the influence of specimen size variations across different studies, the compressive and tensile strengths of the concrete are converted. Specifically, the tensile strength *f_t_* is converted using the formula *f_t_* = 0.395(*f_c_*)^0.55^, as specified in the Chinese concrete code GB 50010-2010 [32]. In addition, the concrete cube compressive strength *f_c_* is converted to the equivalent cylinder compressive strength *f_c_*′ using the relevant conversion coefficients, as detailed in Reference [23].

To enhance the data quality, the original dataset was filtered based on a unified criterion. Specifically, in a set of data with the same test parameters, if the target value Pu of a certain data point differs from the others by more than 15%, while the differences among the remaining data points are within 15%, this data point is identified as an outlier and excluded; under the same test parameter group, if the difference between any two data points in the group exceeds 15%, the whole set of data is removed. After processing, 855 sets of high-quality experimental data were ultimately retained. The database includes the following parameters:

- The material properties: the compressive strength of a concrete cylinder (*f_c_*′) and the elastic modulus of the FRP sheets (*E_f_*);
- The geometrical parameters: the thickness (*t_f_*), width (*b_f_*), and bond length (*L_f_*) of the FRP sheets and the concrete substrate width (*b_c_*);
- The bond strength of the FRP-concrete interface (*P_u_*).

### 2.2. Data Description

**Figure 1:**
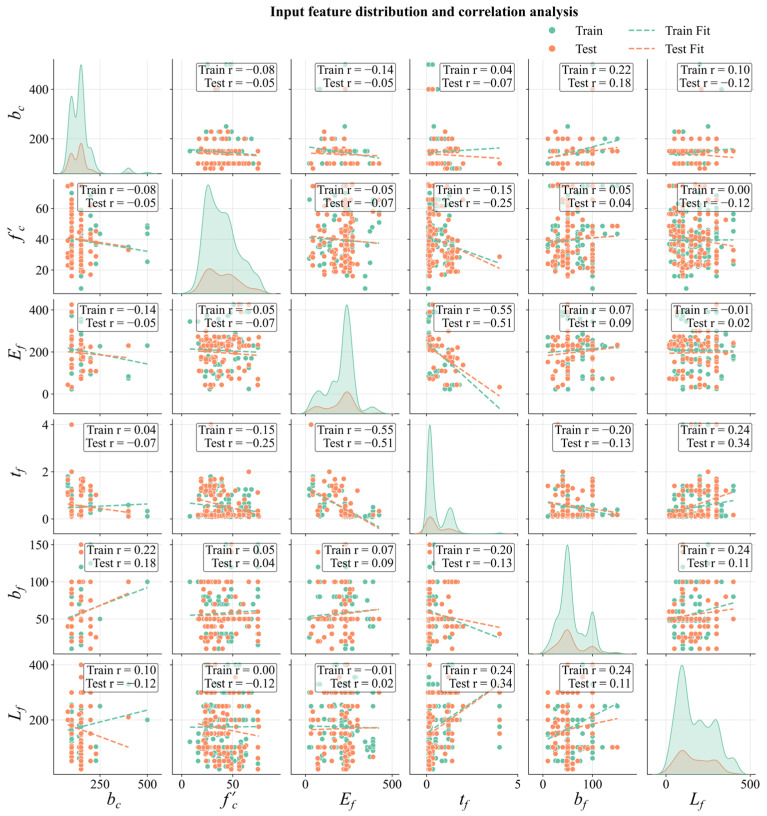
Data distribution and feature correlation analyses.

To develop the predictive model, this study selects material properties (*f_c_*′ and *E_f_*) and geometric parameters (*t_f_*, *b_f_*, *L_f_*, and *b_c_*) as input features, and *P_u_* serves as the target variable. The statistical characteristics of these parameters, including the minimum, maximum, mean, and quartiles (Q1, Q3, etc.), are summarized in Table 2. The dataset is randomly divided into 80% for training and 20% for testing. Figure 1 illustrates the sample distributions in the training and test sets, as well as the correlation analysis of the input features. The results indicate that the training and test sets exhibit a similar distribution in the feature space, and no significant multicollinearity is detected among the input features.

## 3. Methods

### 3.1. Nevergrad Optimization Library

Nevergrad is an open-source, gradient-free optimization library developed by Facebook [33,34]. It efficiently addresses optimization problems in continuous, discrete, or mixed parameter spaces and is well-suited for hyperparameter tuning in machine learning. The library supports multiple optimization algorithms. Seven of these algorithms were selected in this study to tune the hyperparameters of the XGBoost regression model.

1. Covariance matrix adaptation evolution strategy (CMA): The CMA is an evolutionary optimization algorithm based on a multivariate Gaussian distribution [35]. It dynamically updates the mean vector, covariance matrix, and step size parameter to match the geometric characteristics of the objective function. In each iteration, candidate solutions are sampled, and their fitness is evaluated, with the mean updated via weighted recombination. The covariance matrix is refined using both the Rank-μ strategy (current generation information) and the Rank-1 strategy (historical path). An independent evolution path controls the step size scaling, thereby achieving efficient optimization of both the search direction and scale.
2. Two-point differential evolution (TwoPointsDE): TwoPointsDE is a variant of differential evolution whose core innovation lies in replacing the classical binomial crossover with a two-point crossover mechanism [36]. The algorithm randomly selects two crossover points and replaces the parameter segment within the selected interval of the mutation-generated donor vector with the target individual. This design preserves the dependencies between adjacent parameters and reduces the disruption to potentially beneficial schemata, thus enhancing the global exploration capability of the algorithm.
3. Particle swarm optimization (PSO): PSO is a swarm intelligence optimization algorithm that simulates the collective behavior of bird flocks and fish schools [37]. The algorithm is optimized by simulating particles moving through the search space. Each particle represents a potential solution and retains records of its personal best position and the global best position. The PSO dynamically adjusts each particle’s velocity and position based on individual memory and social collaboration, enabling the swarm to progressively converge toward the optimal solution.
4. Random Search: Random Search identifies the optimal solution by performing uniform random sampling of candidate solutions within a predefined search space and evaluating their corresponding objective function values [38].
5. ScrHammersley: ScrHammersley is an optimization algorithm based on low-discrepancy sequences, specifically the Hammersley sequence, combined with scrambling techniques in a quasi-Monte Carlo framework [39,40]. It employs deterministic sampling points to achieve efficient and uniform exploration of parameter space. Compared with a pure random search, ScrHammersley mitigates sample clustering and uneven coverage issues, thereby enhancing the quality of the initial population.
6. DiscreteOnePlusOne: This algorithm is a (1 + 1) evolution strategy variant tailored for discrete optimization within the Nevergrad framework [33,34,41]. It operates through a single-individual iterative optimization mechanism, where each iteration maintains a parent solution and generates an offspring via probabilistic perturbation operators (e.g., discrete parameter flipping or categorical resets). A greedy selection mechanism determines whether the parent solution should be replaced. The algorithm implements an adaptive mutation strategy that dynamically adjusts the perturbation probabilities to maintain the exploration-exploitation balance.
7. NGOpt: NGOpt is a general-purpose optimizer in the Nevergrad library based on dynamic multi-strategy collaboration [33,42]. It is designed to automatically adapt to problem characteristics and select the most suitable optimization strategies.

### 3.2. XGBoost

Extreme gradient boosting (XGBoost), proposed by Chen et al. [43], is an ensemble method based on gradient-boosting decision trees and is widely applied across various domains. The core idea of XGBoost is to integrate multiple weak learners, typically decision trees, using the gradient boosting algorithm [44,45]. In each iteration, XGBoost adds a new tree to fit the residuals (i.e., the differences between the actual and predicted values) of the previous model. The algorithm then updates the model parameters by minimizing the regularized objective function. The objective function of XGBoost is presented in Equation (1).

(1) $$\begin{array}{l} L\left(f\right)=\sum_{i}l\left({\hat{y}}_{i},y_{i}\right)+\sum_{k}\Omega \left(f_{k}\right)\\ \begin{array}{cc} where & \Omega \left(f\right) \end{array}=ϒT+\frac{1}{2}\lambda {\left\|\omega \right\|}^{2} \end{array}$$

where, $l\left({\hat{y}}_{i},y_{i}\right)$ represents the loss function, where ${\hat{y}}_{i}$ is the predicted value, and $y_{i}$ is the actual value. $\Omega \left(f_{k}\right)$ denotes the regularization term, which is used to control the model complexity. $f_{k}$ represents the model of the *k*-th tree, *T* is the number of leaf nodes in the *k*-th tree and ω is the weight of the leaf nodes in the *k*-th tree. Additionally, ϒ represents the penalty regularization term for the leaf nodes, while λ is the penalty regularization term for the leaf weights.

### 3.3. Evaluation Metrics

To quantify the predictive performance of the model, this study employs the mean squared error (MSE), root mean squared error (RMSE), and coefficient of determination (R^2^) as evaluation metrics [46,47,48,49,50,51,52,53]. The formulas for these metrics are as follows:

(2) $$R^{2}=1-\frac{\sum {\left(P_{i}-{\hat{P}}_{i}\right)}^{2}}{\sum {\left(P_{i}-{\bar{P}}_{i}\right)}^{2}}$$

(3) $$\mathrm{RMSE}=\sqrt{\frac{1}{N}\sum_{i=1}^{N}{\left(P_{i}-{\hat{P}}_{i}\right)}^{2}}$$

(4) $$\mathrm{MAE}=\frac{1}{N}\sum_{i=1}^{N}|P_{i}-{\hat{P}}_{i}|$$

where *N* represents the number of samples, $P_{i}$ denotes the actual values, ${\hat{P}}_{i}$ represents the predicted values, and ${\bar{P}}_{i}$ is the mean of the actual values.

### 3.4. Model Construction

**Figure 2:**
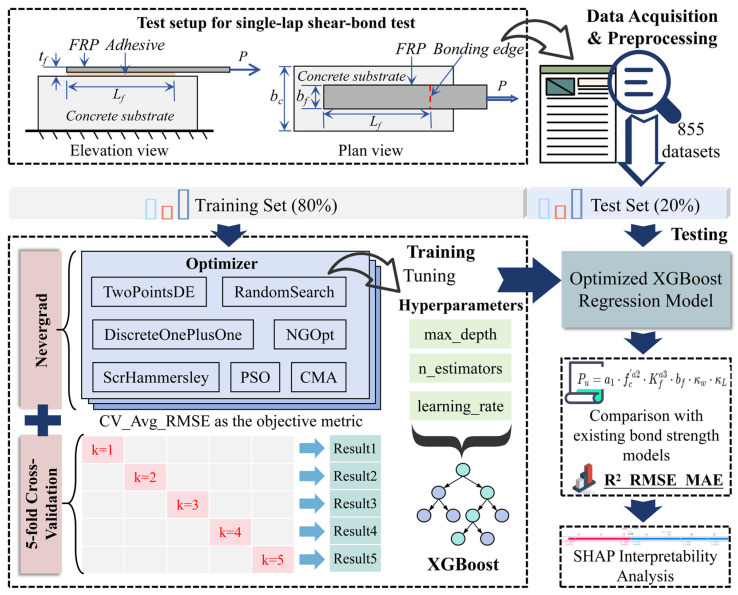
Structure of the XGBoost bond-strength prediction model.

Figure 2 illustrates the framework for constructing the XGBoost bond strength prediction model based on multi-algorithm ensemble optimization. The dataset is randomly split into a training set (80%) and an independent test set (20%). The Nevergrad framework integrates seven optimization algorithms to search for optimal hyperparameters within a predefined search space, including n_estimators, learning_rate, and max_depth. The model uses the five-fold cross-validated average root-mean-square error (CV_Avg_RMSE) as its optimization objective, thereby ensuring robust hyperparameter selection. During optimization, the system records the convergence trajectories and evaluation metrics (R^2^, RMSE, and MAE) of each algorithm. A multi-dimensional algorithmic performance assessment is conducted based on the predictive accuracy of the validation subsets, ultimately selecting the optimal hyperparameter combination. The optimized parameters are then applied to XGBoost to construct a high-performance regression model, which is subsequently validated on an independent test set. A systematic comparison with existing bond strength models is conducted to ensure the superior performance of the constructed model. To further analyze the decision-making mechanism of the model, SHAP analysis is employed to quantitatively characterize the contribution of each input variable to the prediction outcomes.

## 4. Results

### 4.1. Hyperparameter Optimization

**Figure 3:**
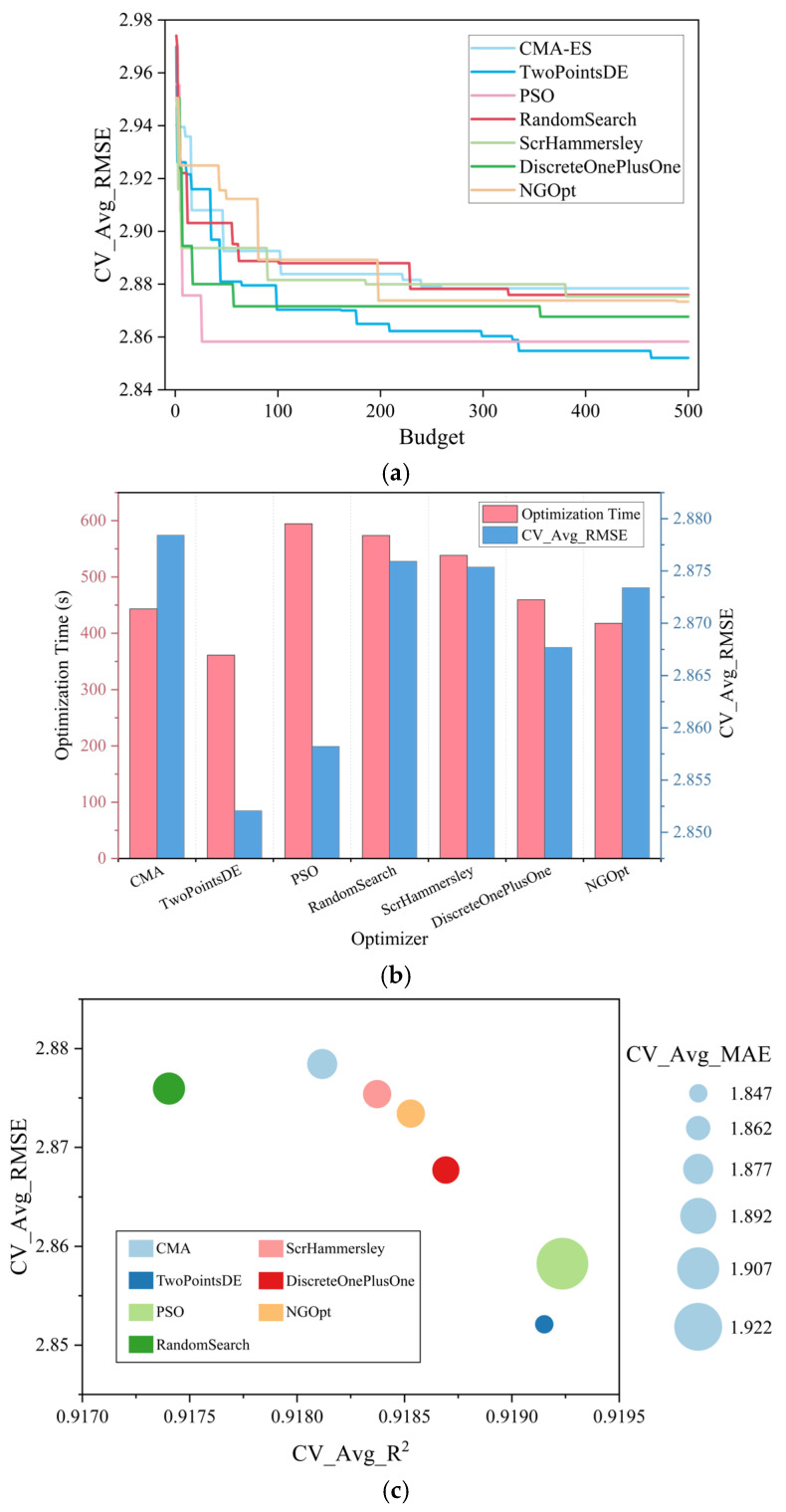

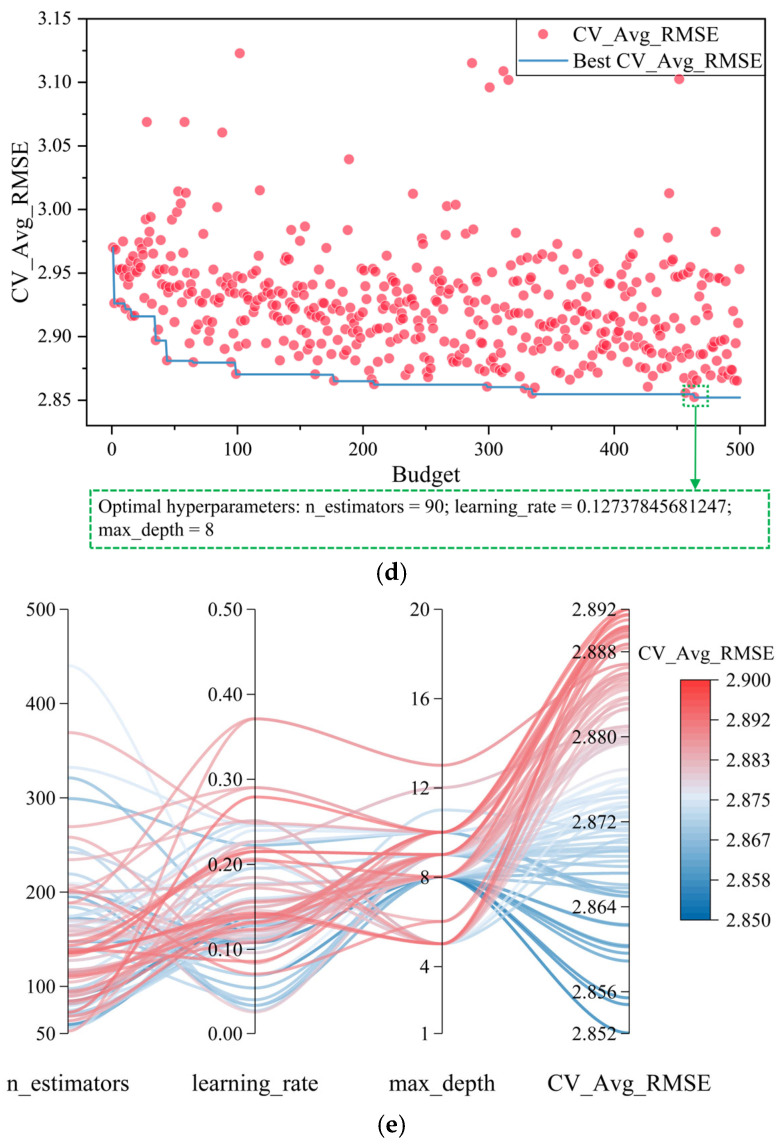
Hyperparameter optimization process of the XGBoost model: (**a**) variation in the best CV_Avg RMSE, (**b**) optimization time of optimizers, (**c**) comprehensive comparison of optimizers’ predictive performance, (**d**) variation in the objective value during the TwoPointsDE optimization process, and (**e**) hyperparameter optimization path of TwoPointsDE (top 100 ranked sets).

The predefined hyperparameter search space is presented in Table 3. Figure 3a presents the variation trend of the objective function value CV_Avg_RMSE with respect to the number of iterations (budget) during the optimization process. Figure 3b shows the best objective values obtained by the seven optimizers, along with their corresponding computational times. Among these optimizers, TwoPointsDE achieves the lowest CV_Avg_RMSE within the 500-iteration budget while requiring the least optimization time. Figure 3c presents a bubble chart that provides an intuitive comparison of the overall performance of the different optimization methods. The horizontal axis represents the cross-validation average R^2^ (CV_Avg_R^2^), and the vertical axis corresponds to CV_Avg_RMSE. The bubble size indicates the cross-validation average MAE (CV_Avg_MAE), and different colors distinguish the optimizers used. From Figure 3c, TwoPointsDE achieves lower CV_Avg_RMSE and CV_Avg_MAE values compared to the other optimizers, while its CV_Avg_R^2^ is second only to PSO, indicating that it exhibits the best overall performance.

Figure 3d,e provides a detailed visualization of the optimization process of TwoPointsDE, illustrating the evolution of the objective function value and the hyperparameter optimization trajectory. Ultimately, TwoPointsDE determines the optimal set of XGBoost hyperparameters as follows: n_estimators = 90, learning_rate = 0.12737845681247, and max_depth = 8. Further details regarding the optimization results for all the optimizers are presented in Table 4.

### 4.2. Comparison of Prediction Performance

**Figure 4:**
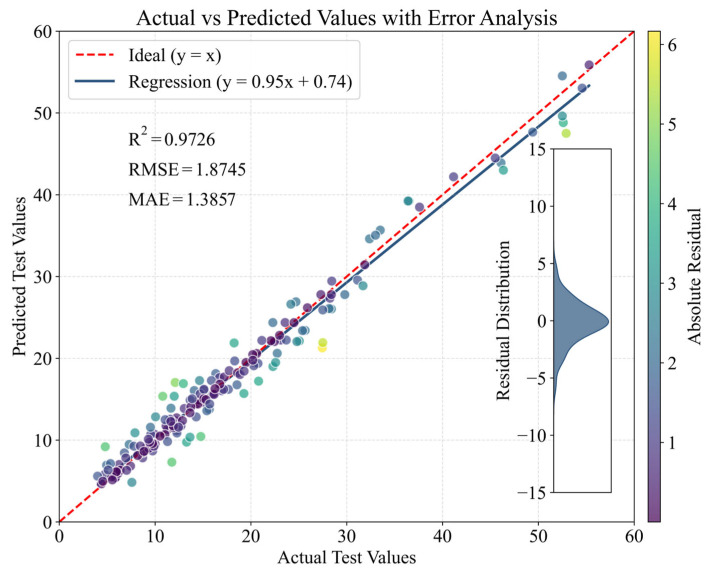
Test set prediction results of the Nevergrad-XGBoost model.

The optimized XGBoost model is used to predict the test dataset, and the results are illustrated in Figure 4. The red dashed line represents the ideal scenario, where the predicted values equal the actual values (y = x), and the blue solid line denotes the regression fit of the model predictions. As shown in the figure, the model achieves an R^2^ of 0.9726, indicating a strong predictive capability. Furthermore, the RMSE and MAE are 1.8745 and 1.3857, respectively, demonstrating low prediction errors and high accuracies. The residual distribution plot on the right reveals that most residuals are symmetrically distributed around zero, with only a few extreme deviations, suggesting no significant systematic bias in the model predictions. The color bar represents the absolute values of the prediction residuals, where darker colors indicate smaller errors and lighter colors correspond to larger deviations. Overall, the optimized XGBoost model performs well on the test dataset, effectively capturing the actual data trends with high predictive accuracy.

**Figure 5:**
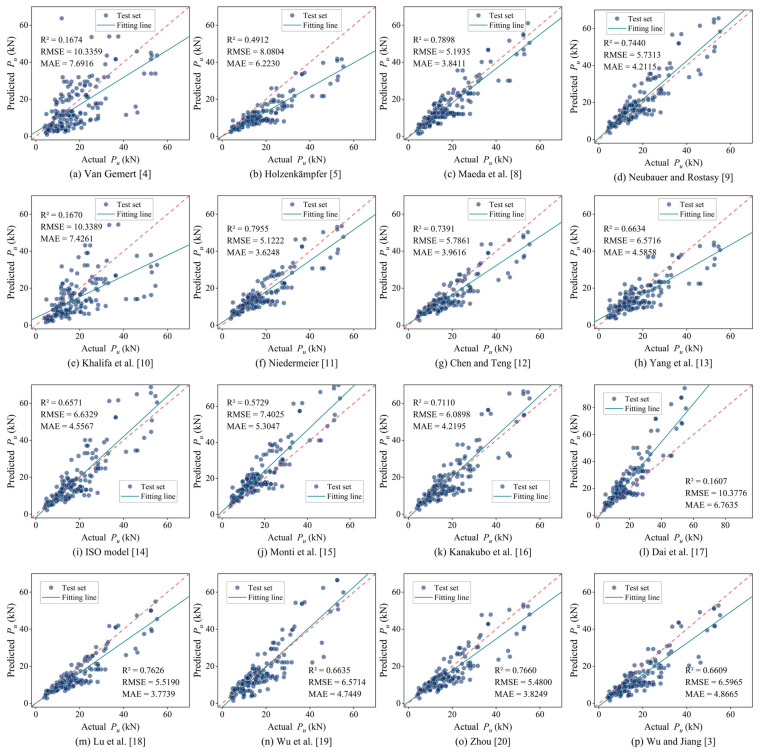
Scatter plot of the prediction results of the existing bond strength models on the test set [3,4,5,8,9,10,11,12,13,14,15,16,17,18,19,20].

To further evaluate the model performance, existing strength prediction models were applied to the same test set, with their prediction results illustrated in Figure 5. The results demonstrate that among the existing models, the model proposed by Maeda et al. [8] (R^2^ = 0.7898, RMSE = 5.1935, MAE = 3.8411), Niedermeier [11] (R^2^ = 0.7955, RMSE = 5.1222, MAE = 3.6248), and Zhou [20] (R^2^ = 0.7660, RMSE = 5.4800, MAE = 3.8249) exhibit relatively superior predictive performance. Additionally, the ANN model trained by Zhou et al. [23] using the same database achieved prediction results of R^2^ = 0.928 and RMSE = 3.584 on the test set. The Nevergrad-XGBoost model developed in this study achieves an approximately 22.3% improvement in R^2^, a reduction of about 63.4% in RMSE, and a reduction of roughly 61.8% in MAE compared to the best-performing existing empirical model (Niedermeier [11]). Moreover, when compared with the ANN model proposed by Zhou et al. [23], the Nevergrad-XGBoost model demonstrates an approximate 4.8% increase in R^2^ and a reduction of about 47.7% in RMSE (see Table 5 for details). These comparisons demonstrate that the proposed model offers markedly superior predictive accuracy and generalization performance.

### 4.3. Interpretability Analysis

This study employs the Shapley Additive exPlanations (SHAP) interpretability analysis method, which is based on cooperative game theory [54,55], to quantitatively assess the feature contributions in the Nevergrad-XGBoost model. By computing the marginal contributions of different feature combinations to the prediction results, this study systematically evaluates the influence mechanisms of various input parameters on the prediction of the bond strength at the FRP-concrete interface. The sign of the SHAP value indicates the directional impact of a feature on the prediction: a SHAP value greater than zero signifies a positive contribution to the prediction outcome, whereas a SHAP value less than zero indicates a suppressive effect.

**Figure 6:**
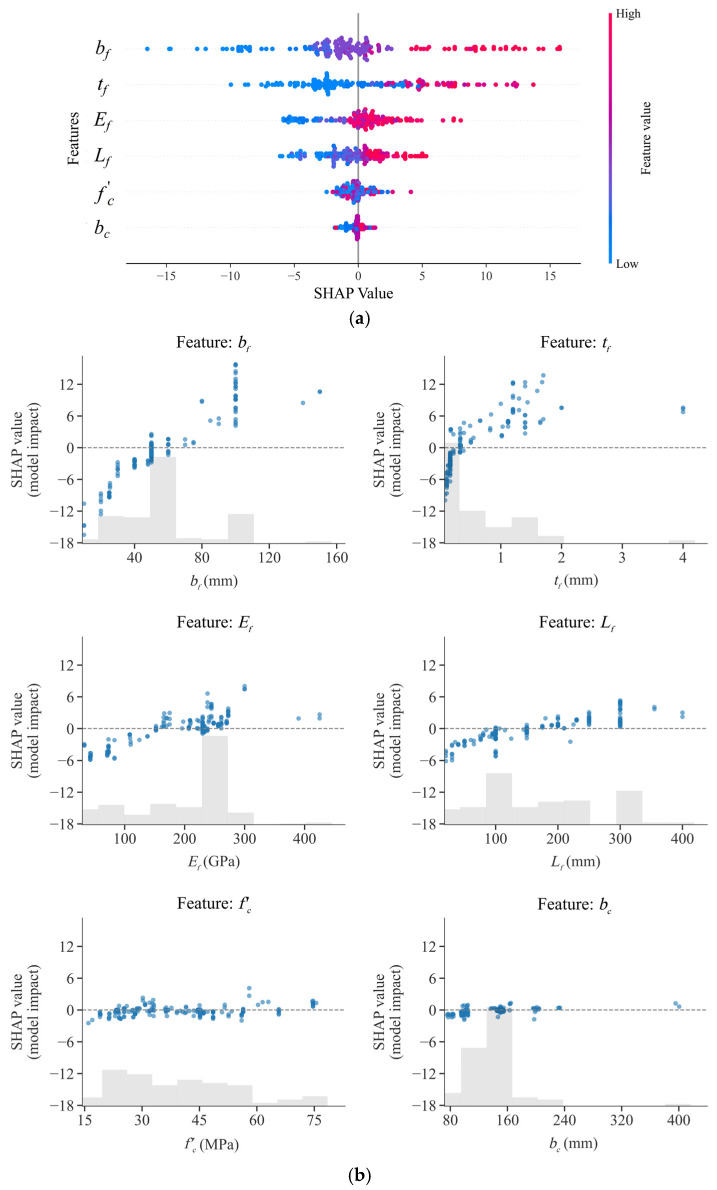
SHAP global explanation: (**a**) SHAP summary plot and (**b**) SHAP scatter plot.

As shown in the SHAP summary plot in Figure 6a, *b_f_*, *t_f_*, and *E_f_* are the three features contributing most to the model output, which is broadly consistent with the feature importance analyses reported by Zhang et al. [25] and Su et al. [26]. Further analysis of the SHAP scatter plots in Figure 6b suggests that the SHAP values of *b_f_*, *t_f_*, *E_f_*, and *L_f_* are generally positively correlated with their respective feature values. Specifically, the pronounced influence of *b_f_* may have arisen from the expansion of the interaction area between the FRP sheet and concrete over a larger surface. An increase in *b_f_* implies an enlarged bond interface area and a more uniform stress distribution, thereby enhancing bond strength. For relatively small *t_f_* values, the SHAP values are negative. This is due to an excessively low *t_f_* causing insufficient axial stiffness (*E_f_t_f_*) of the FRP, localized stress concentrations, and weakened interfacial friction, ultimately reducing the bond strength. Although *L_f_* also exhibited a positive correlation with the SHAP values, its SHAP contributions were mostly confined within the range of [−6, 6]. This may be attributed to the fact that once *L_f_* exceeds the effective bond length, further increasing *L_f_* no longer significantly improves the bond strength. It is noteworthy that numerous studies have demonstrated the significant effect of *b_f_* on bond strength; however, due to differences in interpretability methods, experimental protocols, FRP material types, and adhesive layer properties, the relative importance of each feature may also vary across the literature.

**Figure 7:**
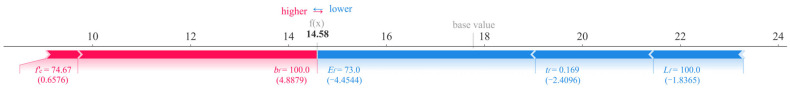
Shap force plot.

Figure 7 presents the SHAP force plot, which intuitively illustrates the specific contributions of individual input features to the final prediction for a particular sample in the test set. According to Table 6, the feature values of this sample are as follows: *b_c_* = 150 mm, *f_c_*′ = 74.67 MPa, *E_f_* = 73 GPa, *t_f_* = 0.169 mm, *b_f_* = 100 mm, and *L_f_* = 100 mm. Based on these feature values, the model’s baseline prediction (i.e., the initial prediction value in the absence of any feature contributions) is 17.7713. For this particular sample, *b_f_* exhibits the highest positive contribution to the prediction, with a SHAP value of 4.8879. In contrast, *E_f_* has the highest negative contribution, with a SHAP value of −4.4544. After aggregating the positive and negative contributions of all features, the final predicted value of the model for this sample is 14.5847 kN. The actual bond strength for this sample is 15.14 kN, demonstrating close agreement between the predicted and actual values. This result validates the effectiveness of the model in predicting the bond strength of the FRP-concrete interface.

SHAP analysis effectively elucidates the influence mechanisms of different features on model prediction outcomes, providing data-driven support for an in-depth understanding of the predictive mechanisms governing the FRP-concrete interfacial bond strength.

## 5. Conclusions

This study proposes a Nevergrad-optimized XGBoost model (Nevergrad-XGBoost) for predicting the bond strength of FRP-concrete interfaces. By integrating seven built-in Nevergrad optimization algorithms with a 5-fold cross-validation mechanism, intelligent hyperparameter search and optimization for XGBoost are achieved, resulting in a high-performance predictive model. The principal findings are summarized as follows:

1. By comparing the hyperparameter optimization results of the seven built-in Nevergrad optimizers, it was found that the TwoPointsDE algorithm achieved the lowest CV_Avg_RMSE (2.85207) within 500 iterations while requiring the shortest computational time (361.25 s), demonstrating an excellent balance between exploration and exploitation. The optimized hyperparameter combination (n_estimators = 90, learning_rate = 0.12737845681247, max_depth = 8) significantly enhanced the predictive performance of the model.
2. The Nevergrad-XGBoost model demonstrates outstanding predictive capability on the test set, with performance metrics of R^2^ = 0.9726, RMSE = 1.8745, and MAE = 1.3857. Compared with the best-performing empirical model, the R^2^ of Nevergrad-XGBoost improves by 22.3%, while the RMSE and MAE decrease by 63.4% and 61.8%, respectively. When compared with the ANN model, the R^2^ increases by 4.8%, and the RMSE decreases by 47.7%, confirming its significant advantages in both predictive accuracy and generalization ability.
3. The SHAP-based interpretability analysis reveals that the contribution of features to the prediction results, from highest to lowest, is as follows: *b_f_*, *t_f_*, *E_f_*, *L_f_*, *f_c_*′, and *b_c_*. The feature values of *b_f_*, *t_f_*, *E_f_*, and *L_f_* show a generally positive correlation with the SHAP values. The global and local interpretation results support the model’s interpretability requirements for engineering applications.

In summary, the XGBoost model optimized by Nevergrad can accurately predict the bond strength at the FRP–concrete interface, providing a new approach for the cost-effective optimization of FRP strengthening parameters. When combined with field monitoring data, the model can support the dynamic evaluation of the residual load-bearing capacity of structures. Moreover, the core algorithmic architecture and interpretability analysis framework employed in this study can be extended to predict the performance of other composite material–concrete interfaces.

## Figures and Tables

**Table 1:** Existing bond-strength models.

Reference	Model
Van Gemert [4]	$P_{u}=0.5b_{f}Lf_{t}$
Holzenkämpfer [5]	$P_{u}=b_{f}\sqrt{G_{f}E_{f}t_{f}}$ $G_{f}=c_{f}f_{t},\;cf=0.204\;\mathrm{mm}$
Tanaka [6]	$P_{u}=\tau_{a}b_{f}L$ $\tau_{a}=6.13-\ln L$
Yoshizawa [7]	$P_{u}=\tau_{a}b_{f}L$ $\tau_{a}=5.88L^{-0.669}$
Maeda et al. [8]	$P_{u}=\tau_{a}b_{f}L_{e},where\;L_{e}=L\;if\;L_{e}>L$ $L_{e}=e^{6.13-0.58\ln(E_{f}t_{f})}$ $\tau_{a}=110.2\times 10^{-6}E_{f}t_{f}$
Neubauer and Rostasy [9]	$P_{u}=\left\{\begin{array}{c} \begin{array}{ccc} 0.64\kappa_{w}b_{f}\sqrt{f_{t}E_{f}t_{f}} & if & L\geq L_{e} \end{array}\\ \begin{array}{ccc} 0.64\kappa_{w}b_{f}\sqrt{f_{t}E_{f}t_{f}}\frac{L}{L_{e}}\left(2-\frac{L}{L_{e}}\right) & if & L<L_{e} \end{array} \end{array}\right.$ $L_{e}=\sqrt{\frac{E_{f}t_{f}}{2f_{t}}}$ $\kappa_{w}=\sqrt{1.123\frac{2-b_{f}/b_{c}}{1+b_{f}/400}}$
Khalifa et al. [10]	$P_{u}=\tau_{a}b_{f}L_{e},where\;L_{e}=L\;if\;L_{e}>L$ $L_{e}=e^{6.13-0.58\ln(E_{f}t_{f})}$ $\tau_{a}=110.2\times 10^{-6}E_{f}t_{f}{\left(\frac{f_{c}^{\prime }}{42}\right)}^{2/3}$
Niedermeier [11]	$P_{u}=\left\{\begin{array}{c} \begin{array}{ccc} 0.78b_{f}\sqrt{G_{f}E_{f}t_{f}} & if & L\geq L_{e} \end{array}\\ \begin{array}{ccc} 0.78b_{f}\sqrt{G_{f}E_{f}t_{f}}\frac{L}{L_{e}}\left(2-\frac{L}{L_{e}}\right) & if & L<L_{e} \end{array} \end{array}\right.$ $L_{e}=\sqrt{\frac{E_{f}t_{f}}{4f_{t}}}$ $G_{f}=c_{f}\kappa_{w}^{2}f_{t}$ $\kappa_{w}=\sqrt{1.123\frac{2-b_{f}/b_{c}}{1+b_{f}/400}}$
Chen and Teng [12]	$P_{u}=\left\{\begin{array}{c} 0.427b_{f}L_{e}\beta_{w}\sqrt{f_{c}^{\prime }}\;if\;L⩾L_{e}\\ 0.427b_{f}L_{e}\beta_{w}\sqrt{f_{c}^{\prime }}\sin\frac{\pi L}{2L_{e}}\;if\;L<L_{e} \end{array}\right.$ $L_{e}=\sqrt{\frac{E_{f}t_{f}}{\sqrt{f_{c}^{\prime }}}}$ $\beta_{w}=\sqrt{\frac{2-b_{f}/b_{C}}{1+b_{f}/b_{C}}}$
Yang et al. [13]	$P_{u}=(0.5+0.08\sqrt{0.01E_{f}t_{f}/f_{t}})\tau_{a}b_{f}L_{e}$ $\tau_{a}=0.5f_{t}$ $L_{e}=100\;\mathrm{mm}$
ISO model [14]	$P_{u}=\left\{\begin{array}{c} \tau_{a}b_{f}L_{e}\;if\;L⩾L_{e}\\ \tau_{a}b_{f}L\;if\;L<L_{e} \end{array}\right.$ $L_{e}=0.125{\left(E_{f}t_{f}\right)}^{0.57}$ $\tau_{a}=0.93f_{c}^{\prime 0.44}$
Monti et al. [15]	$P_{u}=\left\{\begin{array}{c} \;b_{f}\sqrt{\frac{E_{f}t_{f}\tau_{\max}}{3}}\;if\;L⩾L_{e}\\ b_{f}\sqrt{\frac{E_{f}t_{f}\tau_{\max}}{3}}\sin\frac{\pi L}{2L_{e}}\;if\;L<L_{e} \end{array}\right.$ $L_{e}=\sqrt{\frac{E_{f}t_{f}}{\sqrt{4\tau_{\max}}}}$ $\tau_{\max}=1.8k_{b}f_{t}$ $k_{b}=\sqrt{1.5\frac{2-b_{f}/b_{c}}{1+b_{f}/100}}$
Kanakubo et al. [16]	$P_{u}=\left\{\begin{array}{c} 1.1f_{c}^{\prime 0.2}b_{f}L_{e}\;if\;L_{b}>L_{e}\\ \left[0.7\cos\left(\frac{L_{b}}{L_{e}}\right)\pi +1.8\right]f_{c}^{\prime 0.2}b_{f}L_{b}\;if\;L_{b}<L_{e} \end{array}\right.$ $L_{e}=0.7\sqrt{\frac{E_{f}t_{f}}{\sqrt{f_{c}^{\prime 0.2}}}}$
Dai et al. [17]	$P_{u}=\left\{\begin{array}{c} b_{f}\sqrt{2E_{f}t_{f}G_{f}}\;if\;b_{f}<100\;\mathrm{mm}\\ (b_{f}+2\Delta b_{f})\sqrt{2E_{f}t_{f}G_{f}}\;if\;b_{f}⩾100\;\mathrm{mm} \end{array}\right.$ $G_{f}=0.514f_{c}^{\prime 0.236},\Delta b_{f}=3.7\;\mathrm{mm}$ $L_{e}=0.74\sqrt{\frac{E_{f}t_{f}}{f_{c}^{\prime 0.236}}}$
Lu et al. [18]	$P_{u}=\left\{\begin{array}{c} b_{f}\sqrt{2E_{f}t_{f}G_{f}}\;if\;L⩾L_{e}\\ b_{f}\sqrt{2E_{f}t_{f}G_{f}}\frac{L}{L_{e}}\left(2-\frac{L}{L_{e}}\right)\;if\;L<L_{e} \end{array}\right.$ $L_{e}=a_{0}+\frac{1}{2\lambda_{1}}\ln\frac{\lambda_{1}+\lambda_{2}\tan(\lambda_{2}a_{0})}{\lambda_{1}-\lambda_{2}\tan(\lambda_{2}a_{0})}$ $a_{o}=\frac{1}{\lambda_{2}}\sin^{-1}\left(0.99\sqrt{\frac{s_{f}-s_{0}}{s_{f}}}\right),s_{f}=\frac{2G_{f}}{\tau_{\max}}$ $s_{0}=0.0195\kappa_{w}f_{t},G_{f}=0.308\kappa_{w}^{2}\sqrt{f_{t}},\tau_{\max}=1.5\kappa_{w}f_{t}$ $\lambda_{1}=\sqrt{\frac{\tau_{\max}}{s_{0}E_{f}t_{f}}},\lambda_{2}=\sqrt{\frac{\tau_{\max}}{(s_{f}-s_{0})E_{f}t_{f}}}$ $\kappa_{w}=\sqrt{\frac{2.25-b_{f}/b_{c}}{1.25+b_{f}/b_{c}}}$
Wu et al. [19]	$P_{u}=\left\{\begin{array}{c} 0.585b_{f}f_{c}^{\prime 0.1}\kappa_{w}{\left(E_{f}t_{f}\right)}^{0.54}\;if\;L⩾L_{e}\\ 0.585b_{f}f_{c}^{\prime 0.1}\kappa_{w}{\left(E_{f}t_{f}\right)}^{0.54}{\left(\frac{L}{L_{e}}\right)}^{1.2}\;if\;L<L_{e} \end{array}\right.$ $L_{e}=0.395{\left(E_{f}t_{f}\right)}^{0.54}/f_{c}^{\prime 0.09}$ $\kappa_{w}=\sqrt{\frac{2.25-b_{f}/b_{C}}{1.25+b_{f}/b_{C}}}$
Zhou [20]	$P_{u}=\beta_{l}b_{f}\sqrt{2E_{f}t_{f}G_{f}}$ $L_{e}=1.6841\sqrt{\frac{E_{f}t_{f}}{f_{c}^{2/3}}}$ $\beta_{l}=\left\{\begin{array}{c} 1\;if\;L⩾L_{e}\\ \frac{L}{L_{e}}\left(2-\frac{L}{L_{e}}\right)\;if\;L<L_{e} \end{array}\right.$ $\beta_{w}=\sqrt{\frac{2.9-b_{f}/b_{C}}{0.6+b_{f}/b_{C}}}$ $G_{f}=0.0498\beta_{w}^{2}\sqrt{f_{c}^{\prime }}$
Wu and Jiang [3] Lin et al. [21]	$P_{u}(L)=\frac{\alpha }{\beta }E_{f}t_{f}b_{f}\kappa_{L}$ $L_{e}=2\ln\left(\frac{1+\delta }{1-\delta }\right)\cdot \beta$ $\kappa_{L}=\frac{\eta \sqrt{1-\eta^{2}}\sinh(\sqrt{1-\eta^{2}}L/\beta )}{1+\eta \cosh(\sqrt{1-\eta^{2}}L/\beta )}$ $\alpha =0.094f_{c}^{\prime 0.026},\beta =\frac{0.134{\left(E_{f}t_{f}\right)}^{0.5}}{\kappa_{w}f_{c}^{\prime 0.082}}$ $\eta =-3.61e^{-0.4454\frac{L}{\beta }}+4.11e^{-0.3835\frac{L}{\beta }}$ $\frac{\alpha }{\beta }=0.703{\left(E_{f}t_{f}\right)}^{-0.5}f_{c}^{\prime 0.108}\kappa_{w}$ $\kappa_{w}=1+f_{c}^{\prime 0.385}[8{\left(E_{f}t_{f}\right)}^{-0.438}+0.001]\frac{{\left(1-b_{f}/b_{c}\right)}^{0.5}}{1+0.01b_{f}^{1.7}}$ [21]

Note: $P_{u}$ is the bond strength of FRP-concrete interface; $b_{f}$ is the width of FRP sheets; L is the bonded length; $f_{t}$ is the tensile strength of concrete; $G_{f}$ is the fracture energy of interface; $E_{f}$ is the elastic modulus of the FRP sheets; $\tau_{a}$ is the interfacial shear stress; $L_{e}$ is the effective bond length; $t_{f}$ is the thickness of FRP sheets; $b_{c}$ is the width of concrete substrate; $\kappa_{w}$ is the width factor; $f_{c}^{\prime }$ is the compressive strength of concrete.

**Table 2:** Statistical information of the database.

Parameters	Min	Max	Mean	Q1	Median	Q3
*b_c_* (mm)	80	500	144.30	100	150	150
*f_c_*′ (MPa)	8	75.5	39.54	26	36.5	48.56
*E_f_* (GPa)	22.5	425.1	204.80	152.2	230	248.3
*t_f_* (mm)	0.083	4	0.51	0.167	0.169	1
*b_f_* (mm)	10	150	57.52	40	50	70
*L_f_* (mm)	20	400	172.97	100	150	250
*P_u_* (kN)	2.4	56.5	17.80	10.565	15.6	21.955

**Table 3:** Hyperparameter search space for XGBoost.

Hyperparameters	Meanings	Search Ranges
n_estimators	Number of weak learners (decision trees)	[50, 500]
max_depth	Maximum depth of each tree	[1, 50]
learning_rate	Learning rate controls the step size of parameter updates during training	[0.001, 0.5]

**Table 4:** Detailed results of the optimizer hyperparameter optimization.

Optimizer	Optimization Time(s)	n_Estimators	Learning_Rate	Max_Depth	CV_Avg_R^2^	CV_Avg_ RMSE	CV_Avg_ MAE
CMA	443.25	285	0.373695323	32	0.91812	2.87842	1.87640
TwoPointsDE	361.25	90	0.127378457	8	0.91915	2.85207	1.84678
PSO	594.53	212	0.248822202	3	0.91924	2.85821	1.93150
RandomSearch	573.82	175	0.208228973	5	0.91740	2.87593	1.88183
ScrHammersley	538.42	392	0.372326172	13	0.91837	2.87537	1.87154
DiscreteOnePlusOne	459.55	193	0.291644820	10	0.91869	2.86769	1.86871
NGOpt	417.8	348	0.380325336	13	0.91853	2.87340	1.87165

**Table 5:** Comparison of the prediction performance of bond strength models.

Model		R^2^	RMSE	MAE
Empirical or semi-empirical formulas	Maeda et al. [8]	0.7898	5.1935	3.8411
	Neubauer and Rostasy [9]	0.7440	5.7313	4.2115
	Niedermeier [11]	0.7955	5.1222	3.6248
	Chen and Teng [12]	0.7391	5.7861	3.9616
	Kanakubo et al. [16]	0.7110	6.0896	4.2195
	Lu et al. [18]	0.7626	5.5190	3.7739
	Zhou [20]	0.7660	5.4800	3.8249
Machine learning model	ANN (Zhou et al. [23])	0.928	3.584	-
	This paper	0.9726	1.8745	1.3857

**Table 6:** SHAP explanation details for a single sample.

Feature	Feature Value	SHAP Value
*b_c_*	150	−0.0316
*f_c_*′	74.67	0.6576
*E_f_*	73	−4.4544
*t_f_*	0.169	−2.4096
*b_f_*	100	4.8879
*L_f_*	100	−1.8365
Base value	17.7713	
Predicted value	14.5847	
True value	15.14	

## Data Availability

The original contributions presented in this study are included in the article. Further inquiries can be directed to the corresponding author.
